# Energy Underreporting in Low-Calorie and Carbohydrate-Restrictive Diets: Epidemiological Considerations

**DOI:** 10.1016/j.cdnut.2025.107557

**Published:** 2025-09-17

**Authors:** Maximilian Andreas Storz, Alvaro Luis Ronco

**Affiliations:** 1 Department of Internal Medicine II, Centre for Complementary Medicine, Medical Center – University of Freiburg, Faculty of Medicine, University of Freiburg, Freiburg, Germany; 2 Unit of Oncology and Radiotherapy, Pereira Rossell Women's Hospital, Montevideo, Uruguay

**Keywords:** energy intake, diet, low-calorie diet, carbohydrate restriction, nutrition surveys, NHANES

## Abstract

**Background:**

Inaccurate energy intake assessments and dietary underreporting are important barriers to assess reliable health correlates of food consumption in nutritional epidemiology. Studies that do not account for this phenomenon may result in spurious diet–health associations. Whether underreporting occurs more frequently with special diets remains subject to investigation.

**Objectives:**

This study aimed to test the hypothesis whether low-calorie and carbohydrate-restrictive diets were associated with increased odds for energy intake underreporting and investigate whether a lower carbohydrate intake (in %/total energy intake) was associated with a higher discrepancy between self-reported energy intake and total energy expenditure.

**Methods:**

This study used a predictive equation derived from 6497 doubly labeled water measurements to detect erroneous self-reported energy intake in the National Health and Nutrition Examination Surveys (NHANES, 2009–2018). Weighted underreporting prevalence was compared among 3 groups, namely, the United States general population without a special diet, individuals who reported low-calorie diets, and individuals who reported carbohydrate-restrictive diets. Crude and multivariate logistic binomial regression models were built to examine associations between diet and energy intake underreporter status.

**Results:**

Data from 18,150 adult NHANES participants ≥20 y were analyzed. Underreporting occurred almost twice as often in participants reporting low-calorie diets [38.84% (CI: 34.87, 42.95)] and carbohydrate-restrictive diets [43.83% (CI: 33.02, 55.26)] as compared with the general population [22.89% (CI: 21.88, 23.93)]. Both diets were associated with significantly higher odds for underreporting even after an adjustment for sociodemographic factors [odds ratio (OR): 2.32; CI: 1.93, 2.79 and OR: 2.86; CI: 1.85, 4.42, respectively]. Subanalyses in participants denying any weight loss intention/with stable weight revealed a comparable picture. The lowest level of agreement between total energy expenditure and self-reported energy intake was found in carbohydrate-restrictive diets.

**Conclusions:**

Our findings have far-reaching implications, especially with regard to studies that associated carbohydrate restriction or low-calorie diets with favorable health outcomes while not accounting for the herein-suggested phenomena.

## Introduction

Nonaccurate energy intake assessments and energy intake underreporting are major obstacles to assess reliable and trustworthy health correlates of food consumption in the fields of nutrition, dietetics, and epidemiology [[1], [2], [3]]. It is now widely accepted that energy intake underreporting significantly affects the estimates of nutrient intakes [2], potentially leading to false conclusions and misconceptions in nutritional research [4,5].

Although not a novel topic [[1], [2], [3]], recent studies by Bajunaid et al. [4,5] presenting a new predictive equation derived from 6497 doubly labeled water (DLW) measurements to detect erroneous self-reported energy intake, received wide attention in the scientific community. When applying said equation to large and frequently used datasets in nutritional epidemiology (e.g., the National Diet and Nutrition Survey or the NHANES), the authors found that the level of energy intake misreporting exceeded 27% [4,5]. The authors further highlighted that the macronutrient composition from dietary reports was “systematically biased as the level of misreporting increased,” resulting in likely false associations between diet components and BMI [4,5].

In a controversial 2017 editorial, Archer [6] emphasized that dietary data from the United States-based NHANES “are physiologically implausible and inadmissible as scientific evidence”. Almost 8 y later, the improved equation of Bajunaid et al. [4,5] supports this expression of concern and reignites the discourse about dietary data from large-scale epidemiological studies. Although it is of utmost importance to distinguish between the act of underreporting and genuine undereating for the duration of data collection [1], the 2025 articles by Bajunaid et al. [4,5] re-emphasized that underreporting must still be considered a research priority.

Prior work has revealed that underreporting occurs more frequently with regard to unhealthy foods, and is more prevalent among individuals with obesity and females [1]. Whether underreporting affects special diets differently is subject to a controversial debate, particularly when it comes to carbohydrate-restrictive diets, where many adherents mischaracterize their dietary pattern and advocates are often in open disagreement with nutritional authorities [[7], [8], [9], [10]].

In this study, we hypothesized that special diets, including low-calorie diets and carbohydrate-restrictive diets, were associated with increased odds for energy intake underreporting and investigated whether a lower carbohydrate intake (in %/total energy intake) was associated with a higher discrepancy between self-reported energy intake and total energy expenditure (TEE).

## Methods

### Study population: NHANES 2009–2018

To test our hypothesis, we compiled data from the NHANES, a nationally representative, cross-sectional survey of the noninstitutionalized United States population [11,12]. The NHANES provides free data based on a complex, stratified, multistage probability cluster sampling design, ensuring that sample populations are representative of the nation’s noninstitutionalized civilians [[11], [12], [13]]. NHANES data are collected from household interviews and standardized medical examinations. The NHANES recruits ∼5000 participants per annum, and all participants provided written informed consent. The National Center for Health Statistics (NCHS) Research Ethics Review Board reviewed and approved all NHANES survey protocols [14]. For this analysis, data from 5 NHANES cycles were merged to increase the sample size for analysis. To ensure compatibility between the NHANES cycles, this analysis was limited to prepandemic survey cycles.

### Energy intake and carbohydrate intake assessment

Nutrient intake data in this study stem from 2 24-h dietary recalls, from which the mean energy intake (in kcal/d) and carbohydrate intake (in g/d) were calculated. NHANES dietary data were collected using the automated multiple-pass method from the USDA, for which a detailed description may be obtained elsewhere [15]. Participants with an energy intake < 800 kcal/d or >5000 kcal/d were not considered for this analysis.

### Special diet status

As part of the NHANES dietary interview, participants were asked whether they followed a special diet or not [13]. Adherence to a special diet is a self-reported item in the NHANES [13] and was used here to build 3 distinct subpopulations for the analysis: first group of participants who denied any special diets, second group who reported adherence to a low-calorie diet (also referred to as weight loss diets), and, finally, third group reporting adherence to a low-carbohydrate diet. Only participants with reliable dietary data (as determined by the status code “1” in the NHANES variables DR1DRSTZ and DR2DRSTZ) were considered here.

### Estimating TEE and determining underreporter status

TEE was estimated using a predictive equation that was recently presented by Bajunaid et al. [4,5]. The original equation derived from *n* = 6497 DLW measurements reads as follows:

$$\ln\left(\text{TEE}\right)=-0.2127+0.4167\times \ln\left(\text{BW}\right)+0.006565\times \text{Height}-0.02054\times \text{Age}+0.0003308{\text{Age}}^{2}-0.000001852\times {\text{Age}}^{3}+0.09126\times \ln\left(\text{Elevation}\right)-0.04092\times \text{Sex}+0.01940\times A-0.03899\times \text{AA}+0.006238\times \text{AS}+0.02626\times W-0.0155\times H+0.003589\times \text{NA}-0.0006759\times \text{Height}\times \ln\left(\text{Elevation}\right)+0.002018\times \text{Age}\times \ln\left(\text{Elevation}\right)-0.00002262\times {\text{Age}}^{2}\times \ln\left(\text{Elevation}\right)-0.006947\times \text{Sex}\times \ln\left(\text{Elevation}\right)\text{.}$$

Using the equation, one may obtain daily energy expenditure in megajoules per day. Body weight (BW) is entered in kilograms, height in centimeters, and age in years. Sex is coded −1 for males and +1 for females, and the elevation of the measurement location is in meters [4,5]. For the self-reported ethnicity codes, for African individuals living outside Africa, AA was 1 and 0 otherwise; for White, W was 1 and 0 otherwise; for Hispanic, H was 1 and 0 otherwise; for other race/not available, NA was 1 and 0 otherwise [4,5].

To determine whether an individual may be considered an underreporter, one needs to deal with the concept of the 95% predictive interval (PI) associated with this equation. The 95% PI covers a range of values that contains the true value for a single new observation based on specific values of the predictor variables with a likelihood of 95%. Two additional equations are necessary to identify the 95% PI around the prediction [4,5]. The equations for the upper and lower 95% PI are presented hereafter:

Lower95%PI=(pTEE×0.7466)−1.5405

Upper95%PI=(pTEE×1.3395)+2.7668

In both equations, pTEE is the predicted mean TEE (estimated in MJ/d) [4,5]. This interval allows the user to objectively evaluate the confidence that can be placed in any given prediction from the Bajunaid equation. According to the authors, using PIs to screen observations is superior to previous attempts relying on screening dietary reports, which were all based on arbitrary cutoff points [4,5]. Underreporters were herein defined as participants who reported an energy intake below the lower 95% PI:

Underreporter=self-reportedfooddiary-basedenergyintake<lower95%PI

### Special case scenario analysis: deliberate weight loss

The aforementioned TEE equation has been suggested as a good proxy for intake in individuals not attempting to change their weight [4,5]. The desire for weight loss is a special case, as the metabolic rate declines in this scenario [4,5]. It is not inconceivable that individuals selected the examined special diets with an intention to lose weight. In such a scenario, it would be difficult to distinguish between factual reporting errors and true caloric restriction. We thus performed subanalyses to take this particular scenario into account. In a first subanalysis, we only considered NHANES participants who explicitly stated that they did not attempt to lose weight within the last 12 mo. For this, we used variables from the NHANES weight history module. In a second subanalysis, we specifically restricted the analysis to participants with a stable weight over the past 12 mo. Stable weight was defined as either no self-reported weight change within the last 12 mo or a difference (both in terms of gaining or losing weight) of maximally 1 pound in 12 mo.

### Covariates

Covariates in the statistical analysis included age, sex, race/ethnicity, marital status, educational level, smoking status, and the ratio of family income to poverty. The ratio of family income to poverty is an NHANES-specific index that considers the annual family income adjusted for the family size and the poverty threshold guidelines developed by the United States Department of Health and Human Services [16]. The ratio of family income to poverty ranges from 0 to 5 in the NHANES and was categorized into 4 groups for this analysis.

### Statistical analysis

The analytical procedures were performed in accordance with the most recent recommendations on applied survey data analysis by West et al. [17]. A 10-y weight for dietary data was constructed, taking into account the NHANES weighting module tutorial and NHANES analytic guidelines [18]. Weighted survey analyses were performed using Stata’s “svyset” and “svy” commands to account for the complex NHANES survey design. Participants with data missing on any variable of interest were excluded; no imputation procedures were performed.

Sociodemographic, anthropometric, and energy intake data were compared between the general population and 2 groups self-reporting special diets. Data distribution was examined via box plots and subpopulation summary statistics. Normally distributed data were described with the mean and corresponding 95% confidence interval (CI). For categorical variables, we provided weighted proportions with their 95% CIs. The reliability of the estimated weighted proportions was examined using Korn–Graubard intervals based on the user-written “kg-nchs” module in Stata and under consideration of the NCHS reporting standards [19].

Crude and multivariate logistic binomial regression models were built to examine potential associations between diet status and energy intake underreporter status (binary outcome variable, yes compared with no). Odds ratios (ORs) were reported with their 95% CIs. Two different models were constructed: a crude model (model I) and a second multivariate model adjusting for sociodemographic factors (age, sex, race/ethnicity, education level, and income) and smoking status (model II). We then plotted predicted probabilities including their 95% CIs using Stata’s “marginsplot” function [12]. A *P* value < 0.05 was used as the cutoff for statistical significance. All statistical calculations were performed using Stata 18 statistical software (StataCorp. 2023; Stata Statistical Software: Release 18. College Station, TX: StataCorp LLC). Scatterplots and Bland–Altman difference plots (also known as Tukey mean difference plot) were used to visualize the data.

## Results

The final sample analyzed in this study comprised 18,150 adult NHANES participants aged 20 y or older, of which 1611 participants were on a self-reported low-calorie diet and 197 participants were on a low-carbohydrate diet. Supplemental Figure 1 shows a participant inclusion flowchart.

The sample characteristics are displayed in Table 1. Special diet status was not independent of sex, ethnicity/race, or educational level. Individuals who adhered to a self-reported special diet tended to be female and reported a college degree (or above) more frequently. The 3 groups did not vary significantly with regard to age but with regard to BMI. Significant associations were also found with the ratio of family income to poverty. As shown in Table 1, self-reported energy intake varied significantly between groups, with the highest energy intake found in the general population without a special diet [8.94 (CI: 8.86, 9.02) MJ/d] and lowest intake in those reporting a carbohydrate-restrictive diet [7.66 (CI: 7.14, 8.18) MJ/d].

**TABLE 1:** Sample characteristics by diet status.

	General population without special diet (*n* = 16,342)	Participants on a low-calorie diet (*n* = 1611)	Participants on a carbohydrate-restrictive diet (*n* = 197)	*P* value
Sex				< 0.001^1^
Male	49.87% (48.79, 50.96)	38.21% (34.97, 41.56)	45.24% (34.46, 56.49)^∗^	
Female	50.13% (49.04, 51.21)	61.79% (58.44, 65.03)	54.76% (43.51, 65.54)^∗^	
Age (y)	47.16 (46.56, 47.76)	46.49 (44.89, 48.10)	47.55 (44.18, 50.92)	0.686^2^
Ethnicity/race				0.034^1^
Mexican American	8.49% (6.96, 10.31)	7.61% (5.54, 10.35)	3.99% (2.07, 7.55)^∗,†^	
Other Hispanic	5.50% (4.53, 6.65)	5.36% (3.95, 7.23)	4.72% (2.27, 9.55)^†^	
Non-Hispanic White	66.80% (63.55, 69.89)	69.82% (65.92, 73.46)	74.75% (66.81, 81.33)∗	
Non-Hispanic Black	10.89% (9.39, 12.60)	9.84% (8.22, 11.75)	4.76% (2.76, 8.11)∗	
Other race^1^	8.33% (7.35, 9.43)	7.37% (5.98, 9.04)	11.78% (7.23, 18.60)^†^	
Education level				< 0.001^1^
<9th grade	4.13% (3.58, 4.77)	2.33% (1.62, 3.36)	0.88% (0.21, 3.63)^∗,†^	
9–11th grade	9.36% (8.38, 10.45)	7.06% (5.53, 8.97)	0.74% (0.26, 2.04)^∗,†^	
High school graduate/GED	22.78% (21.47, 24.14)	19.13% (15.66, 23.15)	19.81% (11.78, 31.37)	
Some college or AA degree	32.11% (30.69, 33.58)	34.06% (30.16, 38.18)	35.44% (25.50, 46.81)	
College graduate or above	31.61% (29.36, 33.95)	37.42% (32.88, 42.19)	43.13% (31.82, 55.20)^∗^	
Marital status				0.228^1^
Living with a partner/married	63.12% (61.30, 64.92)	67.63% (63.62, 71.39)	63.63% (53.52, 72.67)	
Divorced/separated/widowed	17.66% (16.55, 18.83)	16.42% (14.16, 18.98)	18.00% (11.69, 26.69)	
Never married	19.21% (17.64, 20.89)	15.95% (12.99, 19.43)	18.37% (11.82, 27.42)	
Smoking status				< 0.001^1^
Never smoker	56.28% (54.61, 57.93)	63.26% (59.34, 67.01)	53.81% (41.15, 66.00)^∗^	
Former smoker	24.51% (23.28, 25.78)	26.78% (23.52, 30.31)	29.81% (21.84, 39.24)	
Current smoker	19.21% (17.99, 20.50)	9.97% (7.77, 12.69)	16.37% (9.07, 27.76)^∗^	
Ratio of family income to poverty				< 0.001^1^
<1	14.57% (13.28, 15.96)	9.45% (7.72, 11.52)	10.18% (5.72, 17.47)^∗^	
≥1 and <2	20.63% (19.25, 22.09)	17.99% (15.04, 21.37)	17.90% (11.61, 26.59)	
≥2 and <3	15.14% (14.04, 16.30)	13.19% (11.74, 16.38)	8.07% (4.34, 14.51)^∗,†^	
≥3	49.66% (47.40, 51.93)	58.66% (54.28, 62.92)	63.85% (51.59, 74.53)^∗^	
BMI (kg/m^2^)	28.82 (28.60, 29.03)	32.35 (31.81, 32.88)	31.48 (30.06, 32.89)	< 0.001^2^
Energy intake (kcal/d) (24-h recall based)	2136.96 (2117.99, 2155.93)	1918.40 (1867.20, 1969.59)	1830.51 (1706.06, 1954.95)	< 0.001^2^
Energy intake (MJ/d) (24-h recall based)	8.94 (8.86, 9.02)	8.03 (7.81, 8.24)	7.66 (7.14, 8.18)	< 0.001^2^
Total energy expenditure (equation-based, in MJ/d)	11.22 (11.17, 11.28)	11.54 (11.38, 11.70)	11.73 (11.27, 12.20)	< 0.001^2^
Underreporter				< 0.001^1^
No	77.11% (76.07, 78.12)	61.16% (57.04, 65.11)	56.17% (44.74, 66.98)^∗^	
Yes	22.89% (21.88, 23.93)	38.84% (34.87, 42.95)	43.83% (33.02, 55.26)^∗^	

The table shows weighted proportions and is based on a total number of 18,150 unweighted observations. Continuous variables are shown with their mean and 95% confidence interval. Categorical variables are shown as weighted proportion (95% confidence interval). All weighted proportions can be considered reliable as per the recent National Center for Health Statistics Guidelines, except for those marked with a “†” symbol.

Abbreviations: AA , associate of arts; GED , general equivalency degree.

1 Based on Stata’s design-adjusted Rao–Scott test.

2 Based on regression analyses followed by adjusted Wald tests. The “∗” symbol indicates significant differences in the weighted proportions.

Contrary to these findings, the estimated TEE in MJ/d was highest in those reporting a carbohydrate-restrictive diet [11.73 (CI: 11.27, 12.20)], whereas it was lowest in the general population [11.22 (CI: 11.17, 11.28)]. The weighted proportion of energy intake underreporters was highest in participants self-reporting carbohydrate restriction [43.83% (CI: 33.02, 55.26)] and lowest in the general population [22.89% (CI: 21.88, 23.93)].

**FIGURE 1:**
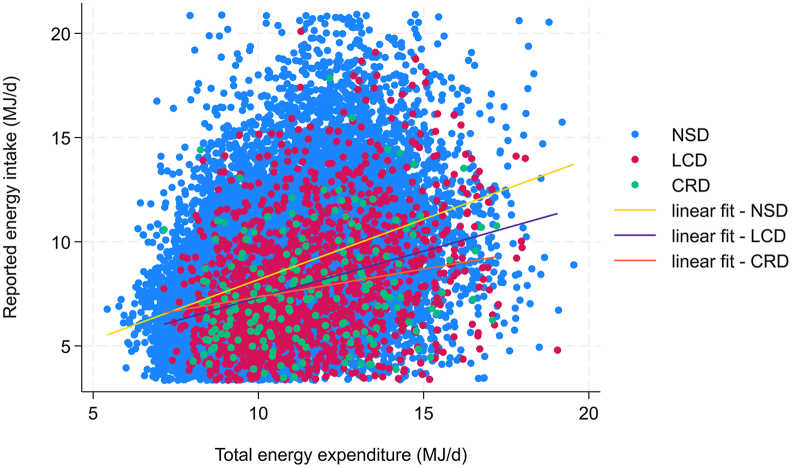
Scatter plot displaying the association between self-reported energy intake and total energy expenditure by diet group. Self-reported energy intake and total energy expenditure are both shown in MJ/d. The golden linear fit line depicts the association for no special diet (NSD) participants, which was highest (*r* = 0.38; *P* ≤ 0.001) among all groups. The weakest correlation was found in carbohydrate-restrictive diet (CRD) participants (*r* = 0.30; *P* = 0.001). Based on 18,150 NHANES participants. LCD, low-calorie diet.

**FIGURE 2:**
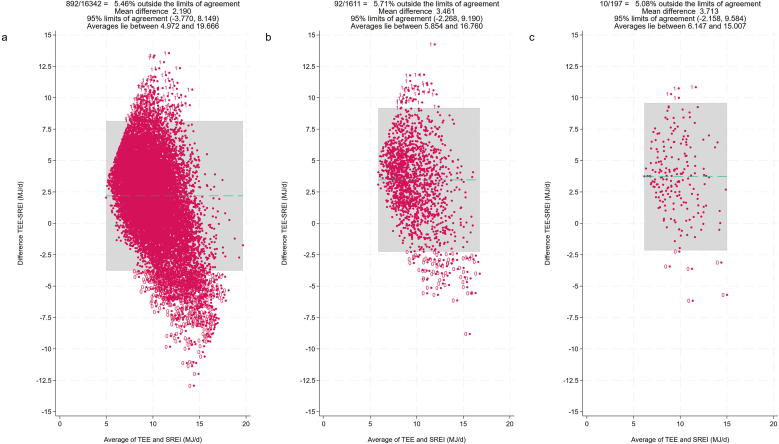
A Bland–Altman difference plot (Tukey mean difference plot) comparing hypothetical levels of agreement between total energy expenditure and self-reported energy intake by diet category. Self-reported energy intake (SREI) and total energy expenditure (TEE) are both shown in MJ/d. (A) Participants who reported no special diet. (B) Participant reporting a low-calorie diet. (C) Participants indicating a carbohydrate-restrictive diet. The lowest level of agreement (e.g., the greatest mean difference between TEE and SREI) was found in those on carbohydrate-restrictive diet. Based on 18,150 NHANES participants.

Figure 1 displays a scatter plot showing the association between self-reported energy intake and TEE by diet group. The strongest correlation (*r* = 0.38; *P* ≤ 0.001) was found in the general population, whereas participants reporting carbohydrate restriction showed the weakest correlation (*r* = 0.30; *P* = 0.001). To further visualize the hypothetical level of agreement between TEE and self-reported energy intake, we constructed diet group-specific Bland–Altman difference plots (also known as Tukey mean difference plot) (Figure 2). The lowest level of agreement (e.g., the greatest mean difference between TEE and self-reported energy intake) was found in those on carbohydrate-restrictive diet. Both figures implied a potential association between diet and underreporter status.

**FIGURE 3:**
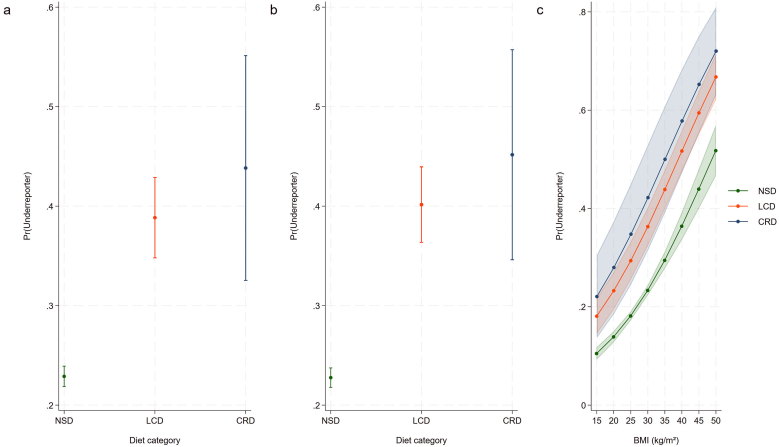
Predicted probabilities of energy intake underreporting depending on diet group based on various multivariate logistic binomial regression models. (A) Crude model. (B) Adjustments for age, ethnicity, sex, education level, and the ratio of family income to poverty were performed. (C) Marginal predicted probabilities of energy underreporting by dietary group based on the same model (+ BMI), illustrating differences depending on BMI. Based on 18,150 NHANES participants. CRD, carbohydrate-restrictive diet; LCD, low-calorie diet; NSD, no special diet.

Subsequently, we built multivariate logistic regression models examining potential associations between diet status and the odds of energy underreporting (Table 2). Two models (a crude model and an adjusted one) were constructed. Even after adjustment for sociodemographic characteristics (model 2), both special diets were associated with significantly increased odds for underreporting. The predicted probabilities of energy intake underreporting depending on diet group based on these models are displayed in Figure 3. In Figure 3C, we additionally adjusted for BMI to investigate potential differences in marginal predicted probabilities of energy underreporting by dietary group depending on BMI.

**TABLE 2:** Crude and multivariate logistic regression models examining potential associations between diet status and the odds of energy underreporting.

Independent variables	OR	CI	*P*	OR	CI	*P*
	Model 1			Model 2		
Diet category						
Omnivore—no special diet	REF	–	–	REF	–	–
Low-calorie diet	2.14	1.78, 2.58	<0.001	2.32	1.93, 2.79	<0.001
Carbohydrate-restrictive diet	2.63	1.66, 4.15	<0.001	2.86	1.85, 4.42	<0.001
Age				0.991	0.987, 0.994	<0.001
Ethnicity						
Mexican American				0.66	0.54, 0.81	<0.001
Other Hispanic				0.75	0.63, 0.89	0.001
Non-Hispanic White				Ref	–	–
Non-Hispanic Black				1.03	0.92, 1.15	0.588
Other race				0.84	0.69, 1.03	0.100
Sex						
Male				REF	–	–
Female				0.75	0.67, 0.84	<0.001
Education level						
<9th grade				1.05	0.84, 1.32	0.653
9–11th grade				0.97	0.79, 1.20	0.786
High school graduate/GED				REF	–	–
Some college or AA degree				0.89	0.77, 1.03	0.112
College graduate or above				0.68	0.57, 0.81	<0.001
Ratio family income/poverty						
<1				1.32	1.13, 1.55	0.001
≥1 and <2				1.25	1.07, 1.46	0.004
≥2 and <3				0.89	0.75, 1.05	0.165
≥3				REF	–	–
Smoking status						
Never smoker				REF	–	–
Former smoker				0.97	0.84, 1.12	0.643
Current smoker				0.95	0.82, 1.09	0.434

The table is based on a total number of 18,150 unweighted observations. Significant regression equations were found for both models: F(2,77) = 39.28 (model 1) and F(17,62) = 14.00 (model 2), respectively, with a *P* value of <0.001 for all models.

Abbreviations: AA , associate of arts; CI , confidence interval; GED , general equivalency degree; OR, odds ratio, REF, reference category.

**FIGURE 4:**
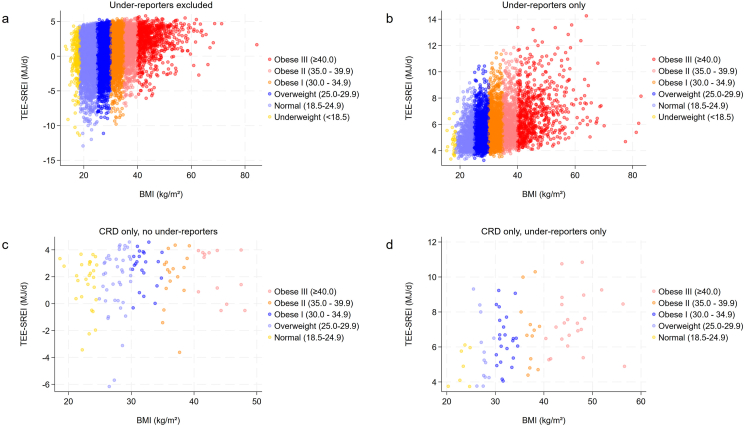
Combined color plots showing the association between the difference of total energy expenditure (TEE) and self-reported energy intake (SREI) and BMI in various subpopulations. The scatterplots depict the association of the hypothetical difference of TEE and SREI with the BMI (kg/m^2^). (A) excludes underreporters, whereas (B) includes underreporters only and shows a significant association between the BMI and the aforementioned TEE-SREI difference (*r* = 0.37; *P* < 0.001). A larger difference (e.g., higher TEE and lower SREI) was associated with a higher BMI. (C) illustrates the low proportion of participants on a carbohydrate-restrictive diet (CRD) with a negative TEE-SREI difference. (D) depicts underreporters only.

Further, we found an association between the difference of TEE and self-reported energy intake and BMI, as shown in Figure 4. A higher (positive) discrepancy between TEE and self-reported energy intake was associated with a higher BMI in non-underreporters (Figure 4A) and underreporters (Figure 4B).

**FIGURE 5:**
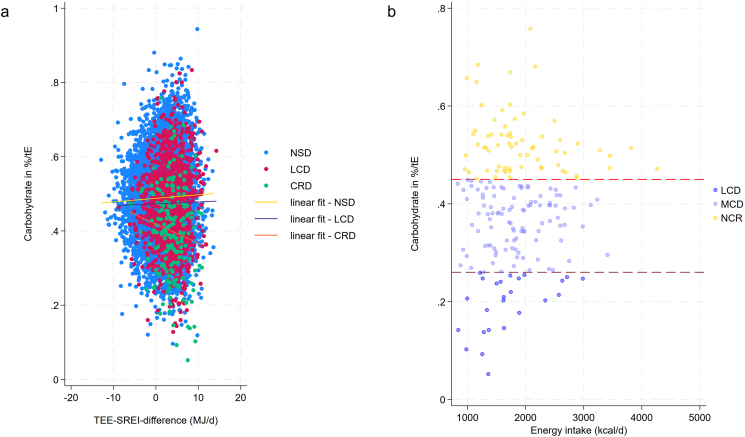
Scatter plot showing the association between the difference of total energy expenditure (TEE) and self-reported energy intake (SER) and carbohydrate intake. The scatterplot in (A) depicts the association between the difference of TEE-SREI and carbohydrate intake expressed as a percentage of total energy (tE) intake. An inverse association was found in those reporting carbohydrate restriction (*r* = –0.29; *P* = 0.025). (B) depicts the carbohydrate intake distribution in those on a self-reported carbohydrate-restrictive diet (CRD). Not all participants met the inclusion of a low-carbohydrate (LCD) or moderate-carbohydrate diet (MCD). Overall, 68 participants did not meet the common low-carbohydrate definitions (marked as NCR, no carbohydrate restriction).

Finally, we investigated whether carbohydrate intake per se (expressed as a % of total energy intake) was associated with a larger discrepancy between TEE and self-reported energy intake. The results are shown in Figure 5A. Carbohydrate intake was negatively associated with said difference in those participants reporting carbohydrate restriction, suggesting that the lower the carbohydrate intake, the larger the discrepancy between TEE and self-reported energy intake. Figure 5B shows an important consideration and potential limitation of this analysis. Not all NHANES participants who self-reported a carbohydrate-restrictive diet also met the common definitions of carbohydrate-restrictive diet. In fact, 68 participants reported a carbohydrate share >46% of total energy intake. This finding implies that although some individuals restrict carbohydrates, they did not automatically fall within the moderate-carbohydrate or low-carbohydrate restriction range (Figure 5B).

In the context of the aforementioned relationship between deliberate weight loss and a lower metabolic rate, we conducted 2 subanalyses. In a first instance, we considered only individuals who explicitly denied any attention to weight loss in the past 12 mo. In this scenario, the OR did not change substantially (Supplemental Table 1), and both special diets remained associated with increased odds for underreporting in the full model. In the second subanalysis, we considered weight changes over the past 12 mo and restricted the regression model to those who reported no weight changes. Again, the OR did not change substantially (Supplemental Table 2).

## Discussion

The present study used NHANES data to examine whether adherence to 2 particular special diets was associated with increased odds for energy intake underreporting. Results suggested that both low-calorie diets and carbohydrate-restrictive diets were associated with significantly increased odds for underreporting even after an adjustment for sociodemographic factors. Although a selection of these 2 diets for weight loss purposes may not be ruled out, subanalyses in individuals explicitly denying weight loss attempts (and with stable weight over 12 mo) revealed a comparable picture. These findings have far-reaching implications, as both diets are among the most popular special diets in the United States [20]. Before the COVID-19 pandemic, from 2007–2008 to 2017–2018, the percentage of adults on any special diet, weight loss or low-calorie diets, and low-carbohydrate diets increased substantially in the United States [20]. Carbohydrate-restrictive diets, for example, are a matter of controversy [9], but currently widely promoted despite a lack of reliable long-term data [21].

As for the NHANES, carbohydrate restriction has been studied extensively with regard to many different endpoints [22,23]. Questions arise, however, as to how much faith one should put into association studies examining a dietary pattern where almost 50% of participants are susceptible to energy intake underreporting. In the aforementioned examples, diet grouping was performed based on self-reported carbohydrate intakes [22,23], which may partly mitigate the aforementioned problem. However, our data suggest that particular caution is warranted when evaluating dietary data from individuals on a special diet. Unlike a Swedish study by Hagström et al. [24], which suggested acceptable agreement of measured energy expenditure and reported energy intake in individuals on a low-carbohydrate diet, our data do not support this finding. Of note, energy intake in the study by Hagström et al. was 2007 (CI: 1570, 2422) kcal/d, which also raises the possibility for weight loss attempts in light of the study participants’ anthropometric and sociodemographic characteristics.

Notably, considerations in this paper are of pure epidemiological and methodological interest and explicitly do not include health outcomes. Using cross-sectional data, it was deemed unfeasible to account for the potential effects of specific food sources (e.g., plant-based compared with animal-based) that are used to replace carbohydrate intake in carbohydrate-restrictive diets [25]. Instead, we focused on energy intake accuracy and energy underreporting odds only. In this context, it is also important to consider that the formerly attributed potential of lower-carbohydrate diets to transiently reduce TEE has been later refuted in an analysis by Hall et al. [26,27]. We believe that this further adds to the call for caution when evaluating the discrepancy between TEE and self-reported energy intake in carbohydrate-restrictive diets.

Our findings have potentially important implications. If additional studies confirm our results, it would be necessary to specifically account for underreporting in all dietary studies involving low-calorie and carbohydrate-restrictive diets; otherwise, this may result in spurious diet–health associations. One hypothetical example is a study that associates low-calorie diets with a certain health outcome based on a lower self-reported energy intake. In case underreporting is involved in a large number of participants, the lower energy intake would not be the decisive mediator. Instead, it would be pertinent to have a look at particular food groups that were substituted for others.

Our analysis is not without limitations. Cross-sectional by nature, our study and the underlying data are potentially subject to recall and reporting bias. Causal inference is not possible. Special diet adherence was self-reported, and the pitfall of dietary pattern mischaracterization has been discussed earlier. Then again, defining groups based on carbohydrate intake (or caloric intake) would have introduced other biases and would not have permitted an analysis of self-reported special diet status. Although 2 dietary recalls have been combined for the analysis in this study, we acknowledge that results could have been different with other dietary tracking and reporting methods (e.g., weighed food diaries). Further, we acknowledge the small sample size in the carbohydrate-restrictive group increasing CIs and uncertainty. Then again, all weighted proportions were meticulously checked for their reliability using “kg_nchs” [19]. The fact that not all self-identified “low-carbohydrate diet” participants were below the “46% of total energy threshold” could be interpreted in 2 ways: said participants either restricted carbohydrates relative to an unknown baseline (but did not achieve a percentage <46% of total energy intake) or said participants misclassified their dietary pattern. Whether the former or the latter is correct remains subject to speculation. The fact that the examined special diets are often selected for weight loss purposes is another limitation in light of the context of a lower metabolic rate in weight loss scenarios. Then again, 2 subanalyses in participants with stable weight/no weight loss intention revealed comparable OR in the fully adjusted models. Finally, we did not consider the activity level of the examined participants for sample size considerations. Including NHANES physical activity data would have further reduced the sample size of the special diet subgroups. It is possible that participants with these special diets are more physically active than others. The equation by Bajunaid et al. [4,5] underestimates TEE in very active individuals (e.g., athletes). However, given the mean BMI of individuals reporting low-calorie diets [32.35 kg/m^2^ (CI: 31.81, 32.88)] and carbohydrate-restrictive diets [31.48 kg/m^2^ (CI: 30.06, 32.89)], we believe that a high proportion of highly active individuals in both groups is unlikely.

Strengths of this analysis include the origin of the data, the state-of-the-art TEE assessment, and the statistical analyses with Tukey mean difference plots. As for future studies, replications with other datasets and other dietary assessment methods are warranted.

Our analysis reiterates that energy intake underreporting is common in nutritional epidemiology and suggests that it occurs more often in individuals on low-calorie or carbohydrate-restrictive diets. These findings have far-reaching implications, especially with regard to studies that associated carbohydrate-restriction or low-calorie diets with favorable health outcomes while not accounting for the herein-suggested phenomena. Resource allocation for studies should take analysis plans into account, and future studies in this field should at least consider the herein presented findings when reporting on the diets under discussion and their associations with health and disease.

## Author contributions

The authors’ responsibilities were as follows – MAS: conceptualization, data curation, formal analysis, resources, software, visualization, and writing – original draft; ALR: supervision; and all authors: project administration, methodology, investigation, validation, writing – review and editing.

## Data availability

NHANES data are publicly available online (https://wwwn.cdc.gov/nchs/nhanes/Default.aspx). The dataset used for this particular study is available from the corresponding author on request.

## Funding

The authors reported no funding received for this study.

## Conflict of interest

The authors report no conflicts of interest.
