# Supplementary material for: Energy Underreporting in Low-Calorie and Carbohydrate-Restrictive Diets: Epidemiological Considerations
Source: Curr Dev Nutr. 2025 Sep 17;9(10):107557. doi: 10.1016/j.cdnut.2025.107557 (PMC12538426; doi:10.1016/j.cdnut.2025.107557)

# Supplementary Materials for

“Energy underreporting in low calorie and carbohydrate-restrictive diets: epidemiological considerations”

by Storz MA and Ronco AL

# Supplementary Tables

## Supplementary Table 1

*Supplementary Figure 1 title*: Crude and multivariate logistic regression models examining potential associations between diet status and the odds of energy underreporting in those who explicitly denied any weight loss intentions in the past 12 months (based on *n* = 9,694 observations)

| Independent variables | OR | CI | *p* | OR | CI | p |
| --- | --- | --- | --- | --- | --- | --- |
|  | Model 1 | | | Model 2 | | |
| Diet Category  Omnivore – no special diet  Low calorie diet  Carbohydrate-restrictive diet | REF  2.55  2.62 | -  1.59-4.11  0.91-7.54 | -  **<0.001**  0.074 | REF  2.79  3.00 | -  1.77-4.41  1.04-8.66 | -  **<0.001**  **0.043** |
| Age |  |  |  | 0.994 | 0.989-0.999 | **0.012** |
| Ethnicity  Mexican American  Other Hispanic  Non-Hispanic White  Non-Hispanic Black  Other Race ^a^ |  |  |  | 0.61  0.96  REF  1.09  0.93 | 0.45-0.83  0.75-1.23  -  0.91-1.31  0.73-1.20 | **0.002**  0.729  -  0.333  0.592 |
| Sex  Male  Female |  |  |  | REF  0.72 | -  0.60-0.87 | -  **<0.001** |
| Education level  Less than 9th grade  9-11th grade  High school graduate/GED  Some college or AA degree  College graduate or above |  |  |  | 1.21  0.97  REF  0.86  0.63 | 0.87-1.68  0.75-1.27  -  0.70-1.05  0.50-0.80 | 0.246  0.845  -  0.145  **<0.001** |
| Annual household income  <20,000$  ≥20,000$ & <75,000$  ≥75,000$ |  |  |  | 1.09  REF  1.05 | 0.91-1.30  -  0.87-1.28 | 0.333  -  0.588 |
| Smoking status  Never smoker  Former smoker  Current smoker |  |  |  | REF  1.01  1.03 | -  0.81-1.25  0.87-1.23 | -  0.964  0.719 |

Supplementary Table 1 legend: Significant regression equations were found for both models: F(2,77) = 7.88 (model 1) and F(16,63) = 11.84 (model 2), respectively, with a p-value of 0.002 for model 1 and a p-value of < 0.001 for model 2. “REF” denotes the reference category. This subpopulation comprises *n* = 9,694 observations, thereof *n* = 189 on a low calorie diet and *n* = 40 on a carbohydrate-restrictive diet. In this sub-analysis, annual household income was selected over the ratio of family income to poverty to maintain an adequate sample size in the carbohydrate-restrictive diet subgroup.

## Supplementary Table 2

*Supplementary Figure 2 title*: Crude and multivariate logistic regression models examining potential associations between diet status and the odds of energy underreporting in those who reported stable weight or weight changes <±1 pound within the last 12 months (based on *n* = 6,145 observations).

| Independent variables | OR | CI | *p* | OR | CI | p |
| --- | --- | --- | --- | --- | --- | --- |
|  | Model 1 | | | Model 2 | | |
| Diet Category  Omnivore – no special diet  Low calorie diet  Carbohydrate-restrictive diet | REF  2.71  2.98 | -  1.90-3.85  1.02-8.70 | -  **<0.001**  **0.045** | REF  2.95  3.12 | -  2.08-4.19  1.15-8.42 | -  **<0.001**  **0.025** |
| Age |  |  |  | 0.987 | 0.982-0.993 | **<0.001** |
| Ethnicity  Mexican American  Other Hispanic  Non-Hispanic White  Non-Hispanic Black  Other Race ^a^ |  |  |  | 0.56  0.53  REF  1.09  0.77 | 0.41-0.76  0.37-0.75  -  0.91-1.31  0.51-1.17 | **0.001**  **<0.001**  -  0.336  0.223 |
| Sex  Male  Female |  |  |  | REF  0.78 | -  0.62-0.96 | -  **0.023** |
| Education level  Less than 9th grade  9-11th grade  High school graduate/GED  Some college or AA degree  College graduate or above |  |  |  | 1.50  1.29  REF  1.00  0.78 | 1.00-2.25  0.92-1.81  -  0.79-1.27  0.59-1.05 | **0.049**  0.133  -  0.997  0.098 |
| Annual household income  <20,000$  ≥20,000$ & <75,000$  ≥75,000$ |  |  |  | 1.30  REF  1.03 | 1.03-1.65  -  0.81-1.31 | **0**.**028**  -  0.797 |
| Smoking status  Never smoker  Former smoker  Current smoker |  |  |  | REF  0.93  0.82 | -  0.73-1.19  0.61-1.10 | -  0.569  0.176 |

Supplementary Table 2 legend: Significant regression equations were found for both models: F(2,77) = 17.86 (model 1) and F(16,63) = 7.95 (model 2), respectively, with a p-value of 0.001 for both models. “REF” denotes the reference category. This subpopulation comprises *n* = 6,145 observations, thereof *n* = 310 on a low calorie diet and *n* = 40 on a carbohydrate-restrictive diet. In this sub-analysis, annual household income was selected over the ratio of family income to poverty to maintain an adequate sample size in the carbohydrate-restrictive diet subgroup.

# Supplementary Figures

## Supplementary Figure 1

*Supplementary Figure 1 title*: Participant inclusion flowchart: the final sample comprised *n* = 18,150 observation


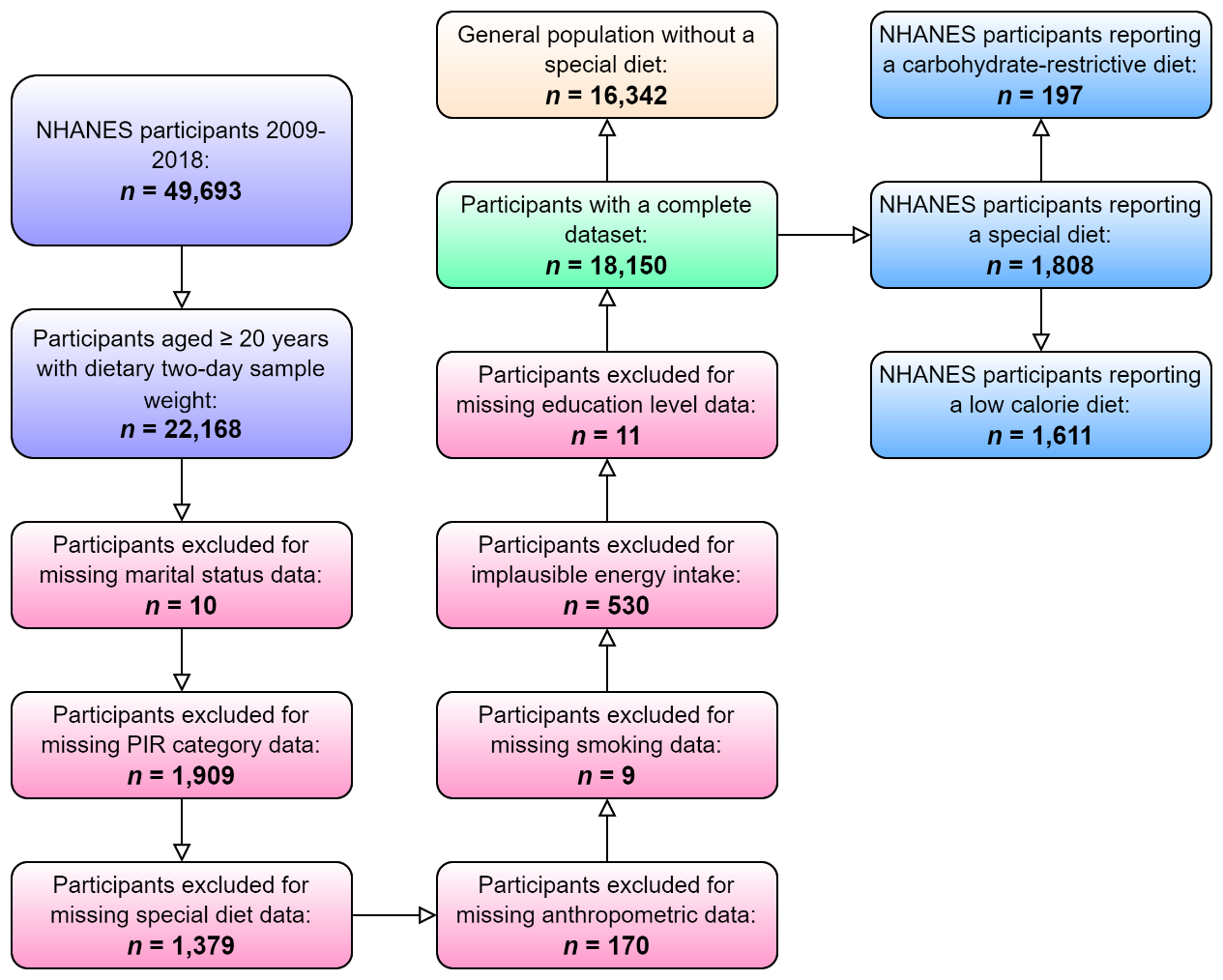

Supplement: Multimedia component 1 [file mmc1.docx]
